# Aquaporin-4’s dynamic expression and biological variations within the tumor obscure the paraneoplastic phenomenon in aquaporin-4-IgG-positive neuromyelitis optica spectrum disorder

**DOI:** 10.1007/s00401-026-03053-y

**Published:** 2026-07-26

**Authors:** Naga Pradyumna Kothapalli, Yong Guo, Nisa Vorasoot, Dean M. Wingerchuk, Eoin P. Flanagan, Divyanshu Dubey, Nanthaya Tisavipat, Fernando X. Cuascut, Hemali Y. Patel, Claudia F. Lucchinetti, Sean J. Pittock

**Affiliations:** 1 Mayo Clinic Health System Neurology and Center for MS and Autoimmune Neurology, Mankato, MN USA; 2 Department of Neurology and Center for MS and Autoimmune Neurology, Mayo Clinic, Rochester, MN USA; 3 Department of Neurology and Center for MS and Autoimmune Neurology, Mayo Clinic, Scottsdale, AZ USA; 4 Division of Neurology, Department of Medicine, Faculty of Medicine, Khon Kaen University, Khon Kaen, Thailand; 5 Department of Neurology, Baylor College of Medicine, Maxine Mesinger Comprehensive Multiple Sclerosis Care Center, Houston, TX USA; 6 Dell Medical School, University of Texas, Austin, USA

Aquaporin-4 (AQP4) expression has been reported in several tumor types in oncologic studies [4, 7, 11, 13]. In patients with aquaporin-4-IgG-positive neuromyelitis optica spectrum disorder (NMOSD), tumor AQP4 expression has been proposed as a potential mechanism underlying paraneoplastic disease [5]. However, prior reports have yielded conflicting results, with some reports showing that AQP4 expression is absent in the associated tumor [1]. Large, systematic evaluations of tumor AQP4 expression within a single, well-characterized cohort of AQP4-IgG-positive NMOSD remain limited. We aimed to determine the frequency and histologic patterns of tumor AQP4 expression in a large cohort of AQP4-IgG-positive NMOSD patients and to assess its relevance to paraneoplastic mechanisms.

We reviewed our database of 400 AQP4-IgG-positive NMOSD patients for coexisting tumors that were not organ-specific, without a specified time limit between tumor and NMOSD onset, as well as for demographic, clinical, radiological, and treatment data (Fig. 1). Pathological evaluation of available tumor tissues was performed on formalin-fixed, paraffin-embedded 5-µm sections stained with the primary antibody to AQP4 (1:250, polyclonal, A5971, Sigma) using the Envision FLEX immunohistochemistry system (Dako). For the negative control, the primary antibody was replaced with 10% fetal calf serum. Normal human brain tissue served as a positive control to verify antibody performance. All slides were independently reviewed by a pathologist blinded to clinical data.

**Fig. 1:**
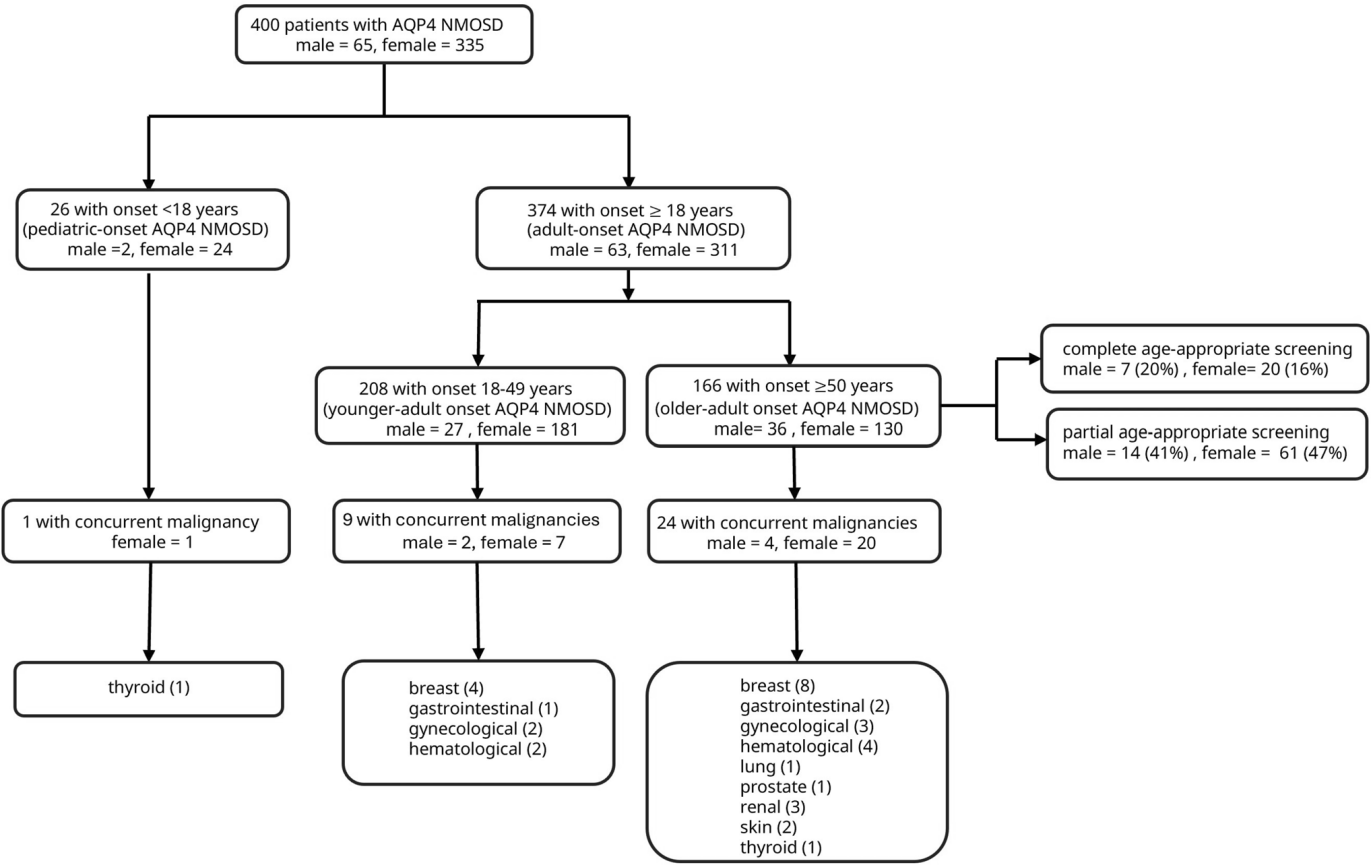
Illustrates the overall demographics of the cohort, including age, sex, and identified concurrent malignancies, as well as the percentage of age-appropriate screenings completed within the older-adult-onset AQP4 NMOSD subgroup, in accordance with American Cancer Society guidelines

In our AQP4-IgG-positive NMOSD cohort, 34 of 400 AQP4-IgG+ NMOSD patients (29 females) had tumors, comprising 8.5% of the cohort. The median age at NMOSD onset in patients with coexisting tumors was 58 years (IQR 47–66). 23 tumors (68%) were identified within 2 years before or after the NMOSD diagnosis. None received immune checkpoint inhibitor therapy. Of 34 patients with malignancy, tumor tissue was available in 13 (38%), of whom 4 (31%) showed AQP4 expression. Except for patient four, the AQP4-positive neoplastic cells displayed epithelial differentiation and potentially secretory functions (Fig. 2). We observed no well-formed lymphoid follicles or germinal center-like tertiary lymphoid structures in the sampled AQP4-positive tumor tissue (Supplementary Fig. 1). Among 16 patients receiving high-efficacy chronic immunosuppressants (12 on Rituximab only, four on Eculizumab, two of whom transitioned from Rituximab), with a median follow-up of 84 months after therapy initiation (IQR 38–96), seven patients (6 on Rituximab) experienced relapses within 1–2 years of tumor detection or recurrence  (Supplementary Tables 1 and 2). No infusion delays or non-compliance were reported. One patient on Eculizumab had a relapse, but compliance and infusion timing is unclear (Fig. 3). Eight patients without relapse started high-efficacy therapies after malignancy remission; one died from tumor progression in 9 months.

**Fig. 2:**
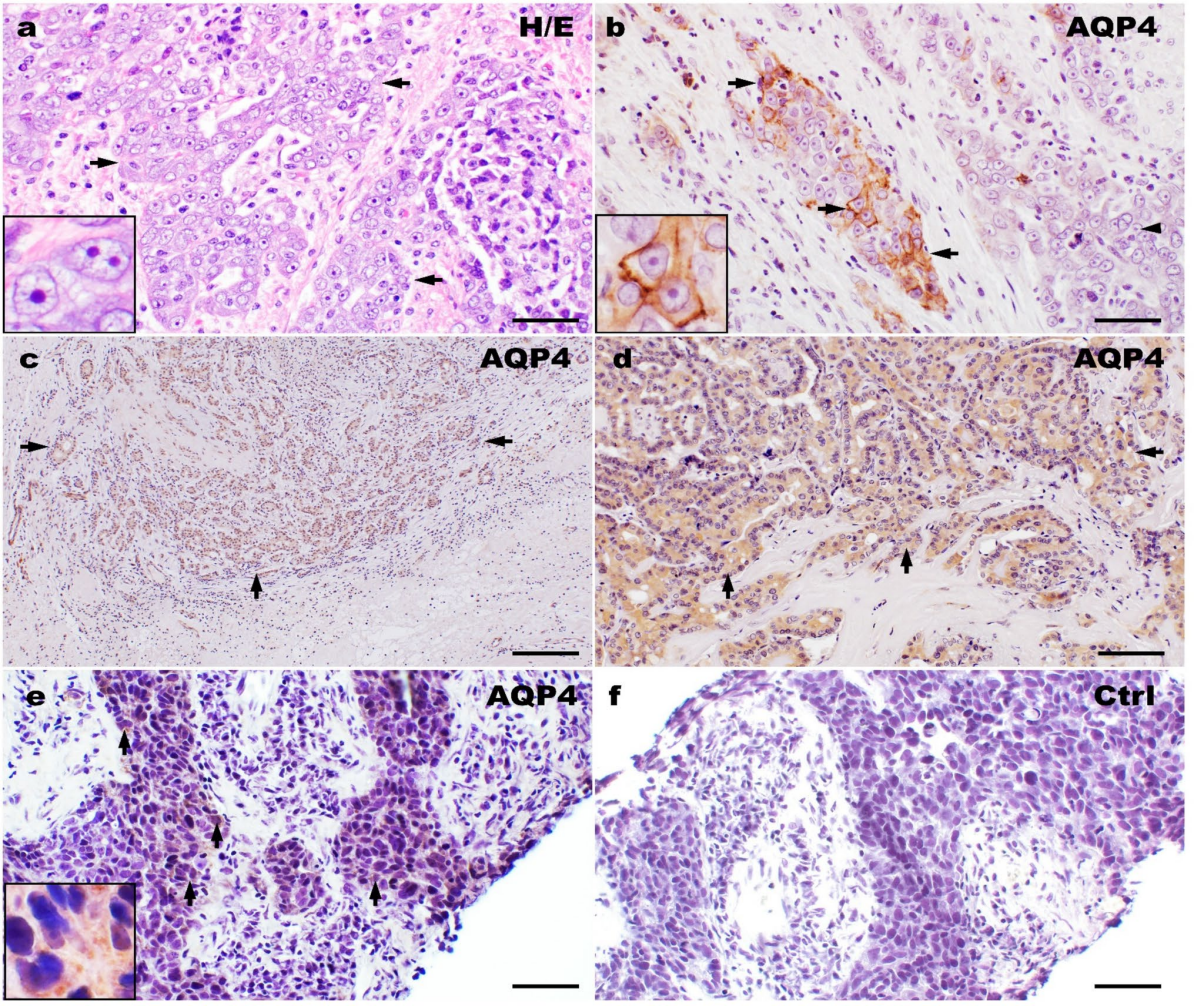
The expression of AQP4 in the tumor tissue of paraneoplastic NMOSD cases. Patient 1: H&E stain of serous carcinoma tissue from the fallopian tube reveals neoplastic cells with large nuclei and prominent nucleoli. The enlarged inset shows prominent nucleoli in neoplastic cells (double nucleoli in the cell on the right). **b** Immunohistochemistry shows variable AQP4 expression in tumor cells, ranging from intense membranous immunoreactivity (marked with arrows and amplified in the inset) to negative staining (indicated with an arrowhead). Patient 3: **c** Resected invasive ductal carcinoma of the breast shows AQP4 immunoreactivity in tumor cells. Patient 2: **d** Thyroid papillary carcinoma shows cytoplasmic AQP4 immunoreactivity (arrows). Patient 4: **e** Immunohistochemistry of a lymph node biopsy from breast cancer shows cytoplasmic AQP4 immunoreactivity in metastatic, poorly differentiated neoplastic cells. **f**  A control stained with 10% fetal bovine serum instead of the primary antibody showed no staining on the consecutive section (**e**). Scale bars: 50 μm (**a**, **b**, **e**, **f**), 200 μm (**c**), 100 μm (**d**)

**Fig. 3:**
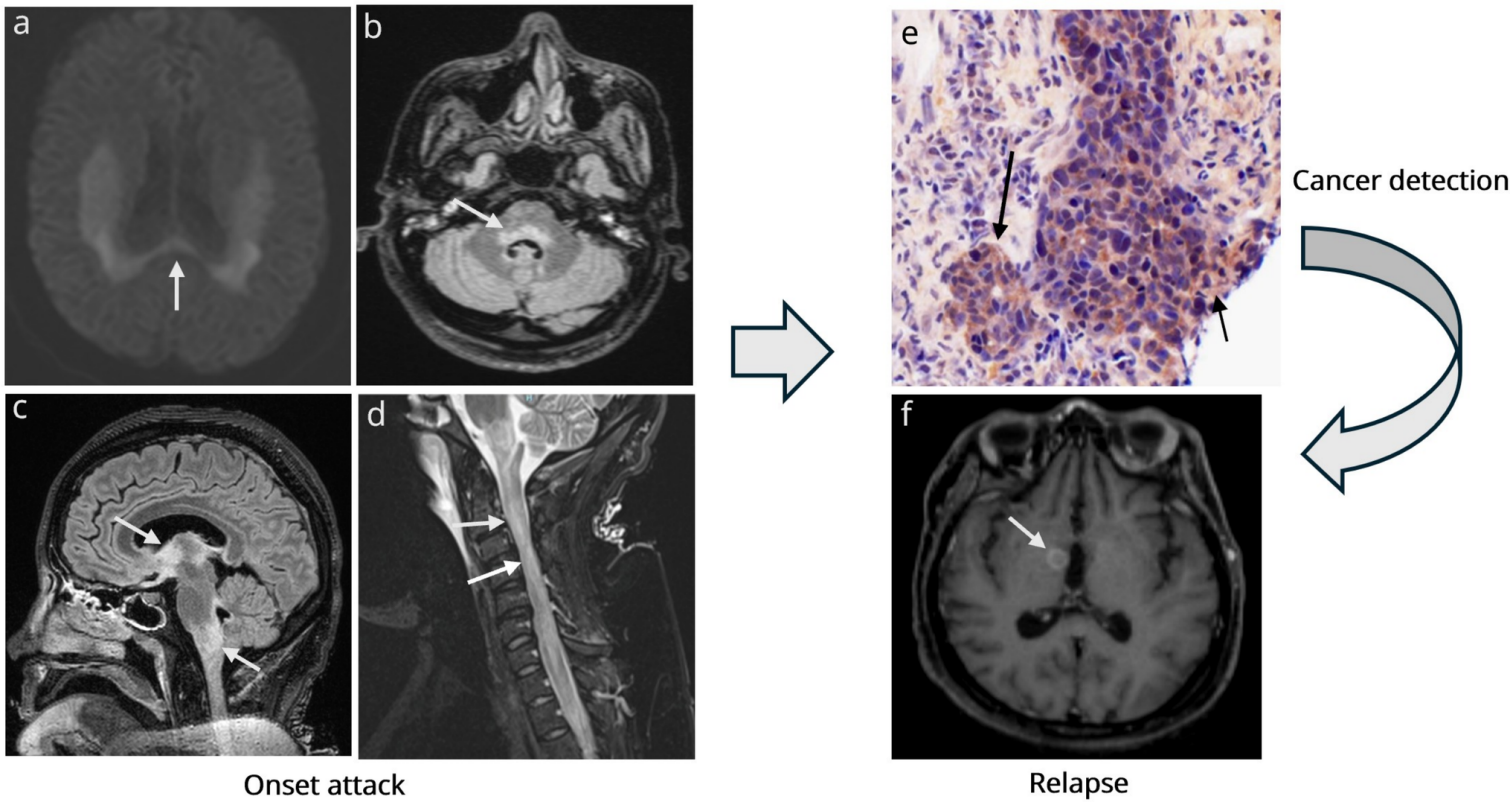
illustrates the case flow of fulminant paraneoplastic AQP4 NMOSD in a 30-year-old female (patient 4) as a timeline (month and year), highlighting the tumefactive lesion at onset and progression (**a**–**d**), tumor diagnosis (**e**), and NMOSD relapse (**f**). **a** Day 2: Tumefactive demyelinating lesion extending across the corpus callosum at onset (diffusion-weighted imaging); **b** Day 10: T2 hyperintensity in the area postrema (FLAIR); Initiated intravenous methylprednisolone and plasma exchange, completing 5 sessions; **c** Day 20: T2 hyperintensity in the hypothalamus and brainstem (FLAIR); **d** Day 27: Long, segmented T2 hyperintensity in the cervical and thoracic cord; progression of the cord lesion while on Eculizumab, initiated on day 21 after NMOSD onset; **e** Cancer diagnosis and aquaporin-4-positive staining (brown) in the breast adenocarcinoma at 3 months after onset attack; **f** Closed ring enhancement in the hypothalamus during relapse, at 6 months after onset attack; information on compliance with Eculizumab infusions at this time point is unclear, but this clearly indicates a highly permeable blood–brain barrier for the inflammatory cascade in the setting of malignancy (intracranial metastasis was excluded with CSF analysis, performed twice during the clinical course). Imaging changes are indicated with white arrows in MRI pictures and with black arrows in histology pictures

In this cohort of AQP4-IgG-positive NMOSD patients with coexisting malignancy, AQP4 expression was predominantly observed in epithelial tumors with secretory differentiation, including ovarian, thyroid, and breast carcinomas, consistent with prior oncologic studies [4, 11, 13]. Tumors in our cohort were identified through age- and risk-factor-based screening or incidentally during screening for other reasons, and approximately 68% were detected within 2 years of NMOSD onset, suggesting a temporal association, and in some instances, relapses were precipitated later in the NMOSD disease course, concomitant with tumor onset or recurrences. Rarely, tumors appeared beyond 2 years due to erroneous screening or incidental discovery. Nevertheless, the lack of AQP4 expression in tumor immunohistochemistry does not preclude a paraneoplastic relationship, as tumor AQP4 expression may be downregulated [14], altered during metastatic dedifferentiation [7], or influenced by biological variability, given that some tumors, such as squamous cell lung carcinomas, exhibit limited yet preserved AQP4 expression solely in acinar cells, unlike adenocarcinomas which display abundant expression [14]. This variability increases the likelihood of missing AQP4 during tissue sampling. Alternative immunologic mechanisms may also contribute to tumor-associated AQP4-IgG-positive NMOSD, including epitope spreading, molecular mimicry, type I interferon-dominant immune signatures in the tumor microenvironment that promote loss of immune tolerance [2], leading to Th-17-predominant cell signatures with altered interferon gamma levels [15], interleukin-6 secretion by tumors during their propagation [8], possible shared HLA class II (DRB1) alleles between NMO and tumors [9], or the HLA class III (C4A) locus and anti-tumor immunity [3]. The inconsistent association with age at onset in NMOSD complicates cancer screening. Yet this may be justified in all late-onset cases, relapses above the age of 50 years, and in relapses on higher-efficacy therapies, including adult and younger-onset cases. Recurrent attacks in tumor-associated AQP4-IgG-positive neuromyelitis optica spectrum disorder (NMOSD), despite high-efficacy therapy such as Rituximab, are mostly due to the persistence of intact AQP4-IgG and an unmitigated Th-17 cell/interleukin-6 cascade in the setting of a tumor, which increases blood–brain barrier permeability, enabling the inflammatory panel to access the central nervous system. This also underscores the pathogenic significance of residual and lifelong AQP4-IgG persistence even during B-cell-depleting therapy [10], possibly due to predominant secretion from long-lasting plasma cells in the bone marrow (CD20-CD19-), which could contribute to NMOSD attacks, particularly in the presence of additional factors, such as tumors, that facilitate breach of immune tolerance, highlighting a lack of need for serum B-cell/CD19+CD20+ repopulation. While the NMOSD lesion progression in the representative case (Fig. 3) may be due to Eculizumab initiation after 21 days from NMOSD onset, as that slightly delayed initiation is associated with a higher odds of poor response in mitigating lesion progression [12], or may suggest the contribution of additional pathological mechanisms, including lytic/antibody-dependent cell-mediated cytotoxicity (ADCC), sub-lytic astrocytopathy [6], and non-lytic/AQP4 receptor modulation, C5 polymorphisms, high-efficacy therapies may influence anti-tumor immunity. Apart from Satralizumab, which could have a relatively favorable effect due to its impact on IL-6 [8] and could limit both tumor progression and blood–brain barrier permeability for AQP4-IgG.

Our study faces limitations including the availability of tumor tissue from cohort patients, challenges in analyzing germinal centers, potential sampling errors caused by heterogeneous AQP4 expression, the lack of a tumor control group without NMOSD, and possible selection bias resulting from non-uniform screening. Larger, multi-institutional studies incorporating proteomic and immune profiling of tumor tissue and serum may further clarify mechanisms linking malignancy and AQP4-IgG autoimmunity and help refine screening strategies for malignancy in NMOSD.

## Supplementary Information

Below is the link to the electronic supplementary material.

Supplementary file1 (DOCX 985 KB)

## Data Availability

The data that support the findings of this study are available from the corresponding author upon a reasonable request.
