# Supplementary material for: Aquaporin-4’s dynamic expression and biological variations within the tumor obscure the paraneoplastic phenomenon in aquaporin-4-IgG-positive neuromyelitis optica spectrum disorder
Source: Acta Neuropathol. 2026 Jul 26;152(1):11. doi: 10.1007/s00401-026-03053-y (PMC13401567; doi:10.1007/s00401-026-03053-y)
Supplement: Supplementary file 1 — Supplementary file1 (DOCX 985 KB) [file 401_2026_3053_MOESM1_ESM.docx]

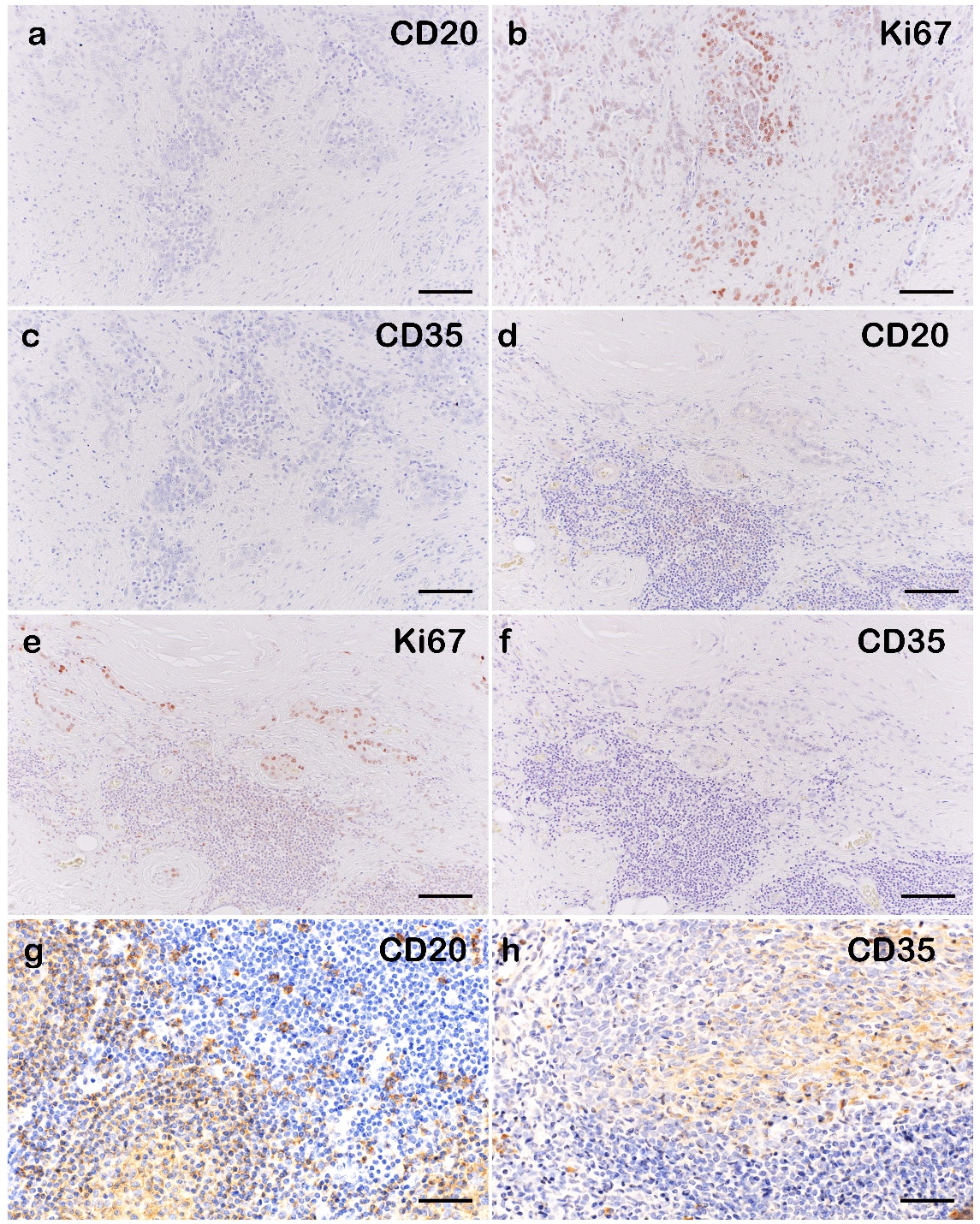


**Supplementary Figure 1. Absence of organized lymphoid follicles or tertiary lymphoid structures in AQP4-positive tumor tissue.**

Additional immunohistochemistry was performed on AQP4-positive tumor tissues using CD20 to identify B-cell aggregates, Ki-67 to assess proliferative activity, and CD35 to detect follicular dendritic cell networks. Representative staining from patient 1 is shown in panels a-c. CD20 staining shows no B cells in the tumor or adjacent stromal tissue (a). Ki-67 labeling is predominantly in neoplastic epithelial cells and scattered infiltrating mononuclear cells, with no evidence of proliferative lymphoid aggregates (b). CD35 staining shows no follicular dendritic cell network (c). Representative staining from patient 2 is shown in panels d-f. At the thyroid carcinoma border, CD20 staining shows no B-cell-rich lymphoid aggregate (d). Ki-67 labeling is predominantly in neoplastic epithelial cells, with no proliferative lymphoid aggregates (e). CD35 staining shows no follicular dendritic cell network (f). Human tonsil tissue was used as a positive control, showing expected CD20-positive B-cell follicles (g) and CD35-positive follicular dendritic cell meshworks (h). Overall, these findings do not support the presence of well-formed lymphoid follicles or germinal center-like tertiary lymphoid structures in the sampled AQP4-positive tumor tissues. Scale bars: 100 μm (a-f), 50 μm (g, h).

| **Pt** | **sex** | **NMOSD Onset age, attack phenotype** | **Inciting event & test that diagnosed malignancy, risk factor (in addition to age) for a specific malignancy if any identified** | **Malignancy** | **tissue expression of AQP4 labelled (Yes/No), PNS-CARE Score** | **NMOSD Onset attack preceding cancer diagnosis (months)** | **Total number of NMOSD attacks within two years of malignancy detection or recurrences** |
| --- | --- | --- | --- | --- | --- | --- | --- |
| 1 | F | 50, unilateral optic neuropathy | Incidentally noted nodule on CXR & smoker | Serous ovarian adeno CA, Stage IV | Yes, 4 | 1 | 2 |
| 2^*^ | F | 59, Short- segmented myelitis | Incidentally noted a thyroid nodule on cervical spine MRI | Papillary thyroid CA | Yes, 6 | 54 | 1 |
| 3 | F | 57, brainstem attack | Routine age-appropriate screening with mammogram | Invasive ductal CA of breast, grade 1 | Yes, 4 | 86^a^ | 0 |
| 4^#^ | F | 30, tumefactive cerebral /brainstem lesion with hypothalamic involvement and longitudinally extensive transverse myelitis | Chest CT performed due to ARDS incidentally revealed lymphadenopathy along with PE leading to biopsy | Poorly differentiated stage IV breast CA | Yes, 6 | 4 | 1 |
| 5 | F | 65, Area postrema syndrome | Odynophagia, weight loss led to CT/PET scans revealing esophageal mass, smoker | Squamous cell CA of esophagus, grade 1 | No, 2 | 0.5 | 2 |
| 6 | F | 70, area postrema syndrome with longitudinally- extensive transverse myelitis | Incidentally noted on pan CT done for myelitis workup, smoker | Clear cell renal CA, grade 2 | No, 2 | 1 | 1 |
| 7 | F | 70, Longitudinally- extensive transverse myelitis | Incidentally noted on PET performed for myelitis workup/morbidly obese | Endometrial adeno CA, grade 1 | No, 2 | 0 | 1 |
| 8 | M | 27, Area Postrema Syndrome with Longitudinally- extensive transverse myelitis | Chest discomfort led to CXR revealing a mediastinal mass | Large B-cell lymphoma | No, 2 | 9 | 1 |
| 9 | M | 66, Longitudinally- extensive transverse myelitis | Information is not available, smoker | Large B-cell lymphoma  Renal cell CA | No, 2  N/A, 2 | N/A | N/A |
| 10^*^ | F | 46, unilateral optic neuropathy | B symptoms & 55 pack years of smoking led to bone marrow biopsy | Hodgkin’s lymphoma stage IV, BM infiltration with recurrences | No, 2 | 24 | 3 |
| 11 | F | 38, unilateral optic neuropathy | Recurrent high grade cervical dysplasia led to hysterectomy which incidentally revealed an endometrial mass, morbid obesity & smoker | Endometrial adeno CA, grade 1 | No, 0 | 163 | 0 |
| 12 | F | 52, Longitudinally- extensive transverse myelitis | Weight loss, viral prodrome led to pan CT revealing lesions in the liver | EBV related B-Cell lymphoma, stage IV | No, 2 | 276 | 0 |
| 13 | F | 54, unilateral optic neuropathy with longitudinally extensive transverse myelitis | Urinary issues led to renal ultrasound  age-appropriate screening with mammogram | Clear cell renal CA, grade 2.  Infiltrating ductal CA of breast, grade 2 | No, 2  N/A, 2 | 169  203 | 1  0 |

**Supplementary Table 1: Characteristics of thirteen AQP4-positive NMOSD patients with coexisting malignancies, examined for Aquaporin-4 expression in tumor tissues.**

BM=bone marrow; CA: carcinoma; Ch=chronic; CT=computed tomography scan; CXR=chest x-ray; EBV=Epstein Barr Virus; F=female; M=male; N=no; N/A=information is not available; PAN CT=computed tomography scan of chest/abdomen/pelvis; PET=whole body positron emission tomography scan; PNS-CARE Score=paraneoplastic neurological syndrome score per 2021 criteria; Pt=patient number; T N/A=tissue not available. (a) The patient exhibited a spiculated mass on a mammogram within two years of NMOSD onset, but further investigation, including a biopsy, was delayed. (*) indicates cases with recurrent attacks despite Rituximab (six patients). (#) The lesion extension in the Eculizumab case description is shown in Figure 3.

| **Pt** | **Sex** | **NMOSD onset age, attack phenotype** | **Inciting event & test that diagnosed malignancy/risk factor (in addition to age) for a specific malignancy** | **Malignancy** | **NMOSD Onset preceding or following cancer diagnosis in months, total number of NMOSD attacks within two years of malignancy detection (or) recurrences** | **PNS-CARE Score** |
| --- | --- | --- | --- | --- | --- | --- |
| 14 | F | 53, Longitudinally- extensive transverse myelitis | nipple discharge led to a skin check, mammogram and then biopsy/none | Paget’s disease of breast, grade 1 | -12, 1 | 6 |
| 15 | F | 47, Longitudinally- extensive transverse myelitis | Age-Appropriate screening by mammogram/none | ductal CA of breast grade 1 | -7, 1 | 6 |
| 16 | F | 60, Longitudinally- extensive transverse myelitis | Incidentally noted on MRI thoracic spine/heavy smoker | Renal cell CA, grade 3 | 4, 2 | 2 |
| 17 | F | 68, Longitudinally- extensive transverse myelitis | No information available | Granulosa ovarian CA | 5, 2 | 2 |
| 18 | F | 15, Area Postrema Syndrome | No information available | Follicular CA of thyroid, grade 1 | 1, 1 | 2 |
| 19 | M | 60, brainstem+ Longitudinally- extensive transverse myelitis | urinary issues were worked up with ultrasound, elevated PSA, biopsy/none | Prostate adeno CA | -17, 1 | 6 |
| 20 | M | 42, Longitudinally- extensive transverse myelitis | abdominal pain, features of bowel obstruction led to CT Abdomen, resection of mass/smoker | colon adeno CA, grade 4 | 312, 0 | 2 |
| 21 | F | 46, bilateral optic neuropathy | upper chest discomfort provoked mammogram led to biopsy of a noted mass /family history of breast cancer | Invasive ductal CA grade 2 | 77, 1 | 6 |
| 22 | F | 59, unilateral optic neuropathy | palpable lump in the breast provoked further work up with biopsy/none | infiltrate breast ductal CA | -77, 0 | 6 |
| 23 | F | 45, Longitudinally- extensive transverse myelitis | palpable breast lump led to mammogram & biopsy/none | invasive Lobular breast CA, grade 2 | 294, 0 | 6 |
| 24 | M | 71, Longitudinally- extensive transverse myelitis + unilateral optic neuropathy | weight loss, thickening of inferior rectus muscle led to workup with biopsy/none | Marginal zone Lymphoma, grade 1 | 3, 1 | 4 |
| 25^*^ | F | 69, Longitudinally- extensive transverse myelitis +UL ON | elevated creatinine led to pelvic ultrasound revealing ovarian masses, led to pan CT and biopsy/none | MALT lymphoma, grade 4 | -5, 2 | 4 |
| 26 | F | 52, Brainstem | information is not available | Breast CA (unknown grading) | -1, 3 | 6 |
| 27 | F | 51, Longitudinally- extensive transverse myelitis | incidentally noted on mammogram screening/smoker | Infiltrating ductal CA, stage IIA | -9, 1 | 6 |
| 28 | F | 71, Longitudinally- extensive transverse myelitis | Skin lesion led to biopsy/none | Squamous cell CA of skin | 120, 0 | 2 |
| 29^*^ | F | 67, Longitudinally- extensive transverse myelitis | skin lesion led to biopsy; has a history of actinic keratosis | Squamous cell CA of skin | 3, 1 | 2 |
| 30 | F | 65, cerebral | incidental on mammogram led to biopsy/smoker | invasive ductal CA, grade 1 | 5, 3 | 6 |
| 31 | F | 66, Longitudinally- extensive transverse myelitis | incidental on mammogram led to biopsy/smoker | ductal CA insitu of breast | 6, 1 | 6 |
| 32 | F | 47, Longitudinally- extensive transverse myelitis | abdominal pain led to workup with CT revealing ovarian mass/none | Ovarian CA,  ductal CA insitu of breast | -40, 0 | 6 |
| 33^*^ | F | 59, Longitudinally- extensive transverse myelitis | Perianal mass led to biopsy/smoker | Anal invasive squamous cell CA, grade 1 | 24, 2 | 2 |
| 34^*^ | M | 71, Longitudinally- extensive transverse myelitis | Extensive smoking history/CT chest and biopsy | Squamous cell CA of lung/grade 3 | 31^b^, 2 | 2 |

**Supplementary Table 2 provides a summary of the characteristics of twenty-one AQP4-positive NMOSD patients with concurrent malignancies. These patients were not analyzed for Aquaporin-4 expression in their tumor tissues due to the unavailability of tissue samples.**

CA: carcinoma; CT=computed tomography scan; F=female; M=male; MRI=magnetic resonance imaging; N/A=information is not available; Pan CT=computed tomography scan of the chest/abdomen/pelvis; PAP=Papanicolaou test; PET=whole-body positron emission tomography scan; PNS-CARE Score=paraneoplastic neurological syndrome score per 2021 criteria; Pt=patient number; Y=yes; ‘-‘ represents the malignancy diagnosis preceding the NMOSD onset and the duration between them. (*) represents cases with recurrent attacks on high-efficacy therapies such as Eculizumab and Rituximab. (b) A lung nodule was noted within a month of NMOSD onset, but the biopsy was initially delayed in favor of observation with monitoring CT scans.

Among 21 patients without tissue samples for AQP4 staining, the diagnosed cancers included breast adenocarcinomas (9), skin squamous cell carcinoma (3), lymphomas (2), ovarian (2), renal (1), thyroid follicular (1), prostate (1), and colon adenocarcinoma (1), lung squamous cell carcinoma (1). These cancers were identified a median of 2 months (range: -77 to 294 months) after NMOSD onset.
